# UV-C Exposure Enhanced the Cd^2+^ Adsorption Capability of the Radiation-Resistant Strain *Sphingomonas* sp. M1-B02

**DOI:** 10.3390/microorganisms12122620

**Published:** 2024-12-18

**Authors:** Yunshi Li, Haoyuan Niu, Shuang Li, Ming Yue, Gaosen Zhang

**Affiliations:** 1 Key Laboratory of Resource Biology and Biotechnology in Western China, Xi’an 710069, China; liyunshi@nwu.edu.cn (Y.L.); lishuang6692@163.com (S.L.); 2 Department of Life Science, Northwest University, Xi’an 710069, China; 3 Key Laboratory of Desert and Desertification, Northwest Institute of Eco-Environment and Resources, Chinese Academy of Sciences, Lanzhou 730000, China; nhy17612400970@163.com; 4 Key Laboratory of Extreme Environmental Microbial Resources and Engineering, Lanzhou 730000, China; 5 Xi’an Botanical Garden of Shaanxi Province, Institute of Botany of Shaanxi Province, Xi’an 710106, China

**Keywords:** *Sphingomonas*, Mount Everest, heavy metal, biosorption, metabolic analysis

## Abstract

Microbial adsorption is a cost-effective and environmentally friendly remediation method for heavy metal pollution. The adsorption mechanism of cadmium (Cd) by bacteria inhabiting extreme environments is largely unexplored. This study describes the biosorption of Cd^2+^ by *Sphingomonas* sp. M1-B02, which was isolated from the moraine on the north slope of Mount Everest and has a good potential for biosorption. The difference in Cd^2+^ adsorption of the strain after UV irradiation stimulation indicated that the adsorption reached 68.90% in 24 h, but the adsorption after UV irradiation increased to 80.56%. The genome of strain M1-B02 contained antioxidant genes such as *mutL*, *recA*, *recO*, and heavy metal repair genes such as RS14805, *apaG*, *chrA*. Hydroxyl, nitro, and etceteras bonds on the bacterial surface were involved in Cd^2+^ adsorption through complexation reactions. The metabolites of the strains were significantly different after 24 h of Cd^2+^ stress, with pyocyanin, L-proline, hypoxanthine, etc., being downregulated and presumably involved in Cd^2+^ biosorption and upregulated after UV-C irradiation, which may explain the increase in Cd^2+^ adsorption capacity of the strain after UV-C irradiation, while the strain improved the metabolism of the antioxidant metabolite carnosine, indirectly increasing the adsorption capacity of the strains for Cd^2+^.

## 1. Introduction

Heavy metals can cause serious harm to the environment due to their non-degradable nature in the environment [1] and also to humans and animals through the food chain [2,3,4]. With the development of the industrial economy, industries such as electroplating, batteries, painting, and mining extraction are the main sources of cadmium pollution in the environment [5,6]. Cadmium (Cd) is a non-threshold toxin that produces toxic effects even at very low concentrations [7]. Excessive amounts of Cd lead to uncontrolled redox reactions in cells and result in the accumulation of reactive oxygen species (ROS). The accumulation of ROS disrupts the cellular structure [8]. The electrochemical method, membrane treatment method, evaporation and concentration method, ion exchange method chemical redox method, and other traditional treatment methods of heavy metal contaminated wastewater have high cost and high energy consumption, easily produce secondary pollution, are difficult for a certain type of heavy metal recycling, and have limited practical application [9,10]. Microbial adsorption has become an effective method suitable for treating heavy metal wastewater at low concentrations (1–100 mg/L), with the advantages of low cost, good effect, short time, reusability, specificity, low risk of secondary contamination, and better feasibility and economy [11,12]. 

Species belonging to the genus *Sphingomonas* have multifaceted functions, such as environmental pollution remediation [13,14], where environmental remediation is mainly based on the degradation of organic matter like dioxins, chlorinated phenols and PAHs [15,16,17]; they can also produce highly beneficial phytohormones, such as sphingan, gellan gum, and indole 3-acetic acid (IAA) [18,19]. Recently, various studies have focused on the physiologic, metabolic, and genetic mechanisms associated with the prolific catabolic capability of *Sphingomonas* [20]. There are few reports on the bioremediation of inorganic compounds such as heavy metals. *Sphingomonas* sp. LK11 and *Sphingomonas* sp. SaMR12 were reported to be tolerant to heavy metal Cd [21,22]. Studies have shown that species of *Sphingomonas* can respond to Cd stress by increasing oxidoreductases (malate dehydrogenase, 2-oxoisovalerate dehydrogenase, 2-oxoisovalerate dehydrogenase, and dihydrolipoyl dehydrogenase); also, Cd accumulation in plants is promoted by increasing glutathione biosynthesis [19,21].

Both UV-C irradiation and Cd stress cause DNA damage and oxidative damage in bacterial cells, and connective pathways may exist for multi-stress resistance [23]. Therefore, microorganisms from the high-UV-C-irradiation environment of the north slope of Mount Everest might also have heavy metal adsorption capability. In this study, we screened the Cd^2+^ adsorption capacity of *Sphingomonas* spp. isolated from Mount Everest, and investigated the difference in Cd^2+^ adsorption of the strains after UV-C irradiation stimulation.

## 2. Materials and Methods

### 2.1. Bacterial Isolation and Cultivation

The species of the genus *Sphingomonas* were isolated from a moraine sample of the north slope of Mount Everest (28.02° N, 86.56° E) on 8 May 2019. Five g of moraine sample was placed in a 50 mL sterile centrifuge tube with 30 mL of sterile saline (0.85%) and resuscitated on a shaker at 180 rpm at 30 °C for 40 min. Hundred µL of solution diluted to one thousandth was then coated to Reasoner’s 2A (R2A) agar medium [24] and incubated at 30 °C for 15 days.

### 2.2. Screening of Cd^2+^ Adsorption Strains

Species of *Sphingomonas*, S2-65, S5-59^T^, M1-B02, S8-45^T^, S9-5^T^, isolated from the north slope of Mount Everest were cultured to the logarithmic phase, respectively, and then prepared as bacterial suspensions, added to CdCl_2_ solution containing Cd^2+^ 100 mg/L, and shaken in a shaker at 30 °C and 160 rpm for 24 h. This was then centrifuged at 6000 r/min for 5 min after shaking, and the supernatant was taken and passed through a 0.22 μm filter membrane; the remaining concentration of Cd^2+^ was determined using hydride generation atomic fluorescence spectroscopy (HG-AFS) [25].

The strain with the highest adsorption of Cd^2+^ was selected. The pH of the CdCl_2_ solution was adjusted to 5, 6, 7, and 8, respectively. The prepared bacterial suspension was added to the CdCl_2_ solution and incubated in a shaker at 30 °C and 160 rpm for 24 h. After being centrifuged at 6000 r/min for 5 min, the supernatant was filtered with a 0.22 μm membrane, and the residual concentration of Cd^2+^ was determined with hydride generation atomic fluorescence spectroscopy (HG-AFS).

### 2.3. Determination of Physiological Characterization

*Sphingomonas* sp. M1-B02 was cultured on R2A liquid medium, TSA liquid medium, NA liquid medium, and PYGV liquid medium to determine the optimal medium. Growth temperature range tests were performed on R2A agar medium in the range of 10–45 °C at 5 °C intervals. The growth pH range was determined on R2A liquid medium at pH 4–12 at intervals of 1. The survival rate after ultraviolet irradiation was determined by culturing the bacterial solution into the logarithmic phase and irradiating it with different doses of ultraviolet radiation. UV-C irradiation induces damage to bacterial DNA by forming pyrimidine dimers, disrupting replication and transcription processes. Additionally, UV-C can cause oxidative stress by generating reactive oxygen species (ROS), which further damages cellular components such as lipids, proteins, and nucleic acids. These effects influence the survival and physiological responses of bacteria, potentially enhancing their stress tolerance mechanisms or altering their metabolic activity. The experimental design considered these impacts to evaluate the survival rates and adaptive responses of the bacteria under UV-C exposure [26].

### 2.4. Genome Sequencing, Assembly, and Annotation

The bacterial isolate was identified based on the 16S ribosomal DNA sequence. The 16S rRNA gene sequencing was identified by Polymerase Chain Reaction (PCR) with universal primers 27F (5′-AGAGTTTGATCCTGGCTCAG-3′) and 1492R (5′-CGGTTACCTTGTTACGACTT-3′) [27]. The genomic DNA of the strain was extracted using the Bacterial Genomic DNA Extraction Kit (OMEGA) and sequenced using the Illumina Hiseq 2000 platform representing >100-fold coverage of the genome. The assembly was performed with the short sequence assembly software SOAPdenovo2 [28]. The completed genome map used the assembly software unicycler version 0.4.8 [29] to assemble the third-generation sequence. Gene annotation was performed by comparing gene sequences with 6 major databases (NR, Swiss-Prot, Pfam, EggNOG, GO, and KEGG) to obtain functional annotation information [30,31,32].

### 2.5. Biological Adsorption Experiment

#### 2.5.1. Preparation of Cell Suspension

A single colony of M1-B02 was taken and inoculated in R2A liquid medium at 160 rpm and 3 °C until logarithmic phase; then, it was centrifuged at 6000 rpm for 5 min, and the supernatant was discarded. The fresh weight of the organism was measured, and the organism was collected, washed 2–3 times with sterile water, and finally resuspended in sterile water and configured into a 0.1 g/mL suspension. 

#### 2.5.2. Adsorption Efficiency Experiment

All adsorption kinetic studies were carried out at a Cd^2+^ concentration of 100 mg/L, pH 7, 30 °C, 160 rpm for 24 h. The measurement time intervals were 10 min, 20 min, 30 min, 40 min, 50 min, 1 h, 2 h, 12 h, and 24 h. The experiments were set up in three groups: Group A was a blank control group without the addition of Cd^2+^; Group B was only exposed to Cd^2+^; and Group C was adsorbed Cd^2+^ after UV irradiation. For Group B, 1mL of prepared suspension and 49 mL CdCl_2_ solution were added to the sterile conical flask. For Group C, 1mL of suspension treated with the optimal ultraviolet radiation dose and 49 mL CdCl_2_ solution were added to the sterile conical flask. When sampling and measuring, these were centrifuged at 6000 r/min for 5 min. The supernatant was passed through a 0.22 μm filter membrane. The remaining concentration of Cd^2+^ in the supernatant was measured using hydride generation atomic fluorescence spectroscopy (HG-AFS), and the adsorption kinetics curve of Cd^2+^ by *Sphingomonas* sp. M1-B02 was drawn. The integrated form of the pseudo-first-order kinetic model and pseudo-second-order kinetic model can be expressed as Equations (1) and (2), respectively [33].

(1) $$q_{t}=q_{e}\left(1-e^{-K_{1}t}\right)$$

(2) $$\frac{t}{q_{t}}=\frac{1}{K_{2}q_{e^{2}}}+\frac{t}{q_{e}}$$

where $q_{e}$ and $q_{t}$ represent the amount of metal ions (mg) adsorbed per unit weight of the adsorbent (g) at equilibrium and at a specific time (t), respectively. $K_{1}$ (1/h) is the pseudo-first-order rate constant, and $K_{2}$ (g/mg/h) is the pseudo-second-order rate constant.

### 2.6. SEM-EDS Analysis

All three experimental Groups A, B, and C were kept at a Cd^2+^ concentration of 100 mg/L, pH 7, 30 °C, 160 rpm for 24 h. When sampling and measuring, the samples were centrifuged at 6000 r/min for 10 min to discard the supernatant, and the precipitate was soaked in 2.5% glutaraldehyde solution overnight. After soaking, the samples were centrifuged again at 6000 r/min for 10 min, and the precipitate was retained. The precipitate was washed three times with 0.1 M PBS buffer (pH 7.0) and then centrifuged at 6000 r/min for 5 min.

Subsequently, the precipitate underwent a dehydration process using a graded ethanol series (50%, 75%, 85%, 90%, 95%, and 100%) for 10 min at each step. After dehydration, the samples were dried using a freeze-drying oven (SCIENTZ-18N, Ningbo, China). The dried samples were mounted on aluminum stubs and coated with a thin gold layer using a sputter coater to enhance conductivity. The microscopic morphology of the bacterial surface was then observed using scanning electron microscopy (SEM) (MIRA3 LMU, TESCAN, Brno, Czech Republic). The elements in the surface precipitate were analyzed using energy dispersive spectroscopy (EDS) (X flash 6130, EDAX Inc., Mahwah, NJ, USA) under these prepared conditions.

### 2.7. Untargeted Metabolomics Analysis of Sphingomonas sp. M1-B02

All three experimental Groups A, B, and C were kept at a Cd^2+^ concentration of 100 mg/L, pH 7.0, 30 °C, and 160 rpm for 24 h. Then, they were centrifuged to remove the supernatant, leaving the bacterium, and six parallel samples were set up for each group. The metabolites were extracted using a 400 µL methanol/water (4:1, *v*/*v*) solution with 0.02 mg/mL L-2-chlorophenylalanin as internal standard. The mixture was allowed to settle at −10 °C and treated by high throughput tissue crusher Wonbio-96c (Wanbo, Shanghai, China) at 50 Hz for 6 min, then followed by ultrasound at 40 kHz for 30 min at 5 °C. The samples were placed at −20 °C for 30 min to precipitate proteins. After centrifugation at 13,000× *g* at 4°C for 15 min, the supernatant was carefully transferred to sample vials for LC-MS/MS analysis. The instrument platform for this LC-MS analysis is the UHPLC-Q Exactive HF-X system of ThermoFisher Scientific (Waltham, MA, USA).

### 2.8. FTIR Spectrometer Analysis

All three experimental Groups A, B, and C were kept at a Cd^2+^ concentration of 100 mg/L, pH 7.0, 30 °C, 160 rpm for 24 h. Then, they were centrifuged at 6000 r/min for 10 min; the supernatant was discarded, and the precipitate was placed in an oven for drying and then ground to powder form. FTIR determination was performed using the KBr compression method [34]. Measurements were performed using Fourier Transform Infrared Spectroscopy (Nicolet, Nexus 870, Madison, WI, USA).

## 3. Results

### 3.1. Screening and Determination of Optimal Adsorption Strain

**Figure 1:**
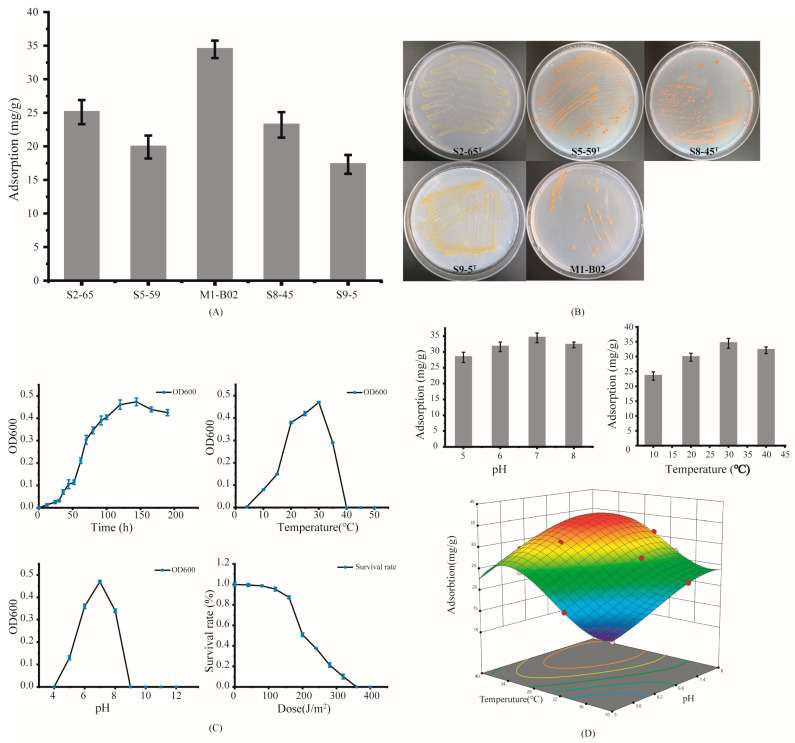
Screening and determination of the adsorption of Cd^2+^ by *Sphingomonas* sp. M1-B02 ((**A**), screening of optimal adsorption strain; (**B**), colonies of the *Sphingomonas* spp.; (**C**), physiological characteristics of *Sphingomonas* sp. M1-B02; (**D**), Optimal adsorption conditions for *Sphingomonas* sp. M1-B02).

The strain with the highest cadmium adsorption capacity in this study was M1-B02, with an adsorption capacity of 34.45 mg/g. The adsorption capacities of strains S2-65, S5-59^T^, S8-45^T^, and S9-5^T^ were 25.1, 19.9, 23.2, and 17.3 mg/g, respectively, which are much lower than M1-B02 (Figure 1A,B). *Sphingomonas* sp. M1-B02 could grow in a temperature range of 10–35 °C (optimum, 30 °C), at pH ranging from pH 5.0 to 8.0 (optimum, pH 7.0), and the survival rate of the strain exceeded 99% at a UV-C irradiation intensity of 80 J/m^2^ (Figure 1C). The growth of the strain was monitored by measuring the optical density at 600 nm (OD600), which reflects cell density by quantifying light scattering caused by the cells in suspension.

Temperature and pH are important factors affecting adsorption [25]; temperature affects the equilibrium capacity of the adsorbent and the adsorption efficiency, and increasing the temperature increases the rate of diffusion of the adsorbent in the external boundary layer and the internal pores of the adsorbent particles. At acidic pH levels, the attraction between the adsorbent and metal cations decreases [35]. The adsorption capacity of M1-B02 for Cd^2+^ was different with different pH. With pH 7, the highest adsorption capacity was 34.45 mg/g, with pH 5 being only 82% of the maximum adsorption capacity, pH 6 being 91%, and pH 8 being only 93% (Figure 1D). The reason for this change may be that as the pH increases, the charged carboxyl, amino, and hydroxyl groups on the cell surface begin to be exposed, enhancing the binding of Cd^2+^ to the adsorption sites [36]. The decrease in adsorption at a pH of 8 may be due to the precipitation of metal hydroxides under alkaline conditions [37,38]. The effect of temperature on the adsorption of Cd^2+^ by the strain was also more significant, and the temperature was proportional to the adsorption amount below 30 °C. The adsorption amount of the strain at 40 °C was lower than that at 30 °C.

The kinetic study of Cd^2+^ biosorption by *Sphingomonas* sp. M1-B02 was carried out using the pseudo-first-order kinetic model and pseudo-second-order kinetic model. The adsorption kinetics models were plotted as the relationship between $q_{t}$ and t, and the values of correlation coefficients (R^2^), $q_{e}$, and $K_{x}$ are shown in Table 1. Here, $K_{x}$ represents the rate constant for the respective kinetic model (pseudo-first-order or pseudo-second-order).

**Figure 2:**
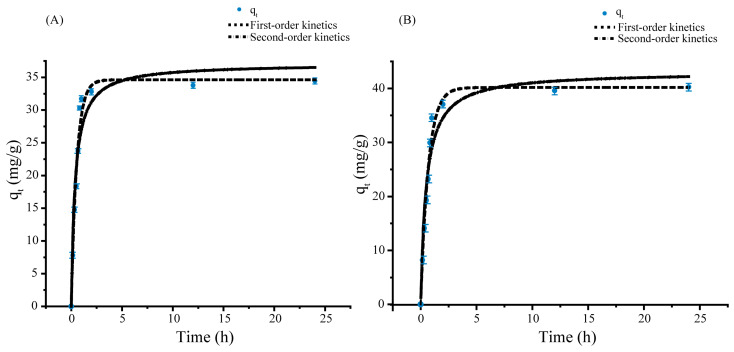
Biosorption dynamic curves of *Sphingomonas* sp. M1-B02 ((**A**), Direct adsorption of Cd^2+^; (**B**), adsorption of Cd^2+^ after UV stress).

The fitting results are shown in Figure 2. The adsorption of Cd^2+^ by strain M1-B02 at the initial Cd^2+^ concentration of 100 mg/L belonged to the rapid adsorption stage in 0–2 h, and after 3 h, the adsorption reached the equilibrium state. The unit adsorption amount of Cd^2+^ by strain M1-B02 at equilibrium was 34.45 mg/g, and the remaining concentration of Cd^2+^ in the solution was 31.10 mg/L, and the adsorption rate reached 68.90%. The adsorption of Cd^2+^ by strain M1-B02 after UV stress belonged to the rapid adsorption stage in 0–2 h, and reached the equilibrium state after 3 h. The unit adsorption of Cd^2+^ by strain M1-B02 at equilibrium was 40.28 mg/g; the residual Cd^2+^ concentration in the solution was 19.44 mg/L, and the adsorption rate reached 80.56%.

### 3.2. Identification of a Novel Species Sphingomonas sp. M1-B02^T^

Strain M1-B02 was purified and cultured on R2A agar medium for 72 h; colonies were circular with regular margins, convex, and yellow. Strain M1-B02 consisted of Gram-negative, aerobic, non-motile, non-spore-forming, and rod-shaped cells (1.4–3.0 µm × 0.4–0.6 µm). Species found to be closely related to strain M1-B02 were *Sphingomonas soli* NBRC 100801^T^, *Sphingomonas asaccharolytica* NBRC 15499^T^, *Sphingomnas panacisoli* HKS19^T^ with 16S rRNA gene sequence similarity levels of 98.65%, 98.44%, and 98.01%, respectively. The neighbor-joining, maximum-likelihood, and minimum-evolution phylogenetic trees (Figures S1–S3) based on 16S rRNA showed M1-B02, and *S. soli* NBRC 100801^T^ formed a stable branch. The genome size of *Sphingomonas* sp. M1-B02 was 3,605,070 bp with an N50 value of 3,605,070 bp and with a guanine–cytosine (GC) content of 65.63 mol%. The total number of coding sequencings (CDSs) in *Sphingomonas* sp. M1-B02 genome was 3488, and the number of RNA was 48, including 45 tRNAs and one set of 5S rRNA, 16S rRNA, and 23S rRNA (Table S1). No plasmid was presented in *Sphingomonas* sp. M1-B02. The ANIb, ANIm, Ortho ANI (Average Nucleotide Identity), and AAI (Average Amino acid Identity) values between strain M1-B02 and the related strains were all lower than the 95% threshold defined by prokaryotes [39,40]. The dDDH values between the strain M1-B02 and other *Sphingomonas* species were from 14.10% to 35.30%, which were also lower than 70%, the threshold defined by prokaryotic species (Table S2). The major fatty acids of strain M1-B02 were Summed Feature 8 (58.7%), C_14:02_-OH (13.5%), Summed Feature 3 (10.3%), and C_16:0_ (6%); polar lipids of strain M1-B02 included diphosphatidylglycerol (DPG), phosphatidylglycerol (PG), phosphatidylethanolamine (PE), four unidentified phospholipids (PLs), two unidentified glycolipids (GLs), four unidentified lipids (L), and sphingoglycolipid (SGL), which were similar to those of other *Sphingomonas* spp. (Figure S4).

The full-length 16S rRNA gene sequencing and genome data of *Sphingomonas* sp. M1-B02 were stored in DDBJ/EMBL/GenBank with accession numbers PP130124 and CP110679, respectively.

### 3.3. Effect of Cd^2+^ on Sphingomonas sp. M1-B02

**Figure 3:**
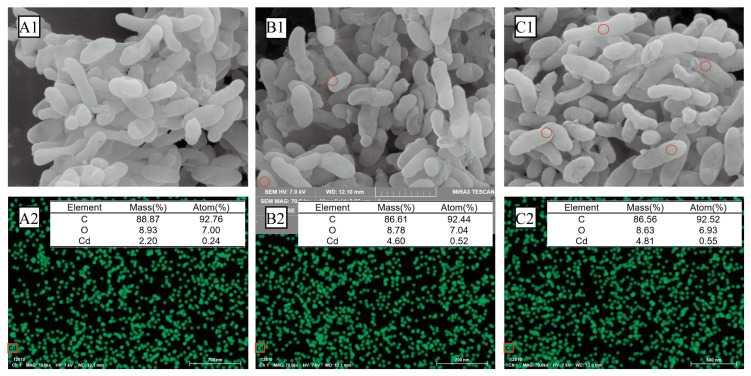
Electron microscopy comparison of strain M1-B02 before and after Cd^2+^ adsorption. (**A1**,**B1**,**C1**) Scanning electron microscopy (SEM) images of strain M1-B02 under different conditions. Red circles highlight regions of Cd^2+^. (**A2**,**B2**,**C2**) Energy-dispersive X-ray spectroscopy (EDS) analysis of corresponding samples showing the elemental composition. The table summarizes the mass percentage (Mass%) and atomic percentage (Atom%) of carbon (C), oxygen (O), and cadmium (Cd). The bottom images in (**A2**,**B2**,**C2**) map the spatial distribution of Cd^2+^ (green dots) on the bacterial surface.

SEM-EDS analysis verified the presence of Cd^2+^ on the surface of the bacterium, and the results are shown in Figure 3A–C. In Figure 3A1, the SEM image shows the rod-shaped morphology of strain M1-B02 in Group A before Cd^2+^ adsorption, with a relatively smooth surface. The corresponding EDS data in Figure 3A2 indicate the elemental composition of the bacterial surface, with a Cd mass percentage of 2.20%, confirming the initial presence of Cd on the surface. After Cd^2+^ adsorption, as shown in Figure 3B1, the surface of strain M1-B02 became slightly roughened, and visible precipitates appeared on the surface. EDS analysis (Figure 3B2) confirmed that these precipitates contained Cd, with the Cd mass percentage increasing to 4.60%. This indicates the successful adsorption of Cd^2+^ onto the bacterial surface. In Group C, where strain M1-B02 was exposed to UV-C stress before adsorption, the SEM image in Figure 3C1 shows a similarly roughened surface with more abundant precipitates compared to Group A. EDS analysis (Figure 3C2) demonstrated a further increase in the Cd mass percentage to 4.81%, suggesting enhanced adsorption capacity under UV-C stress conditions. The results clearly indicate that the surface morphology of strain M1-B02 changes after Cd^2+^ adsorption, with Cd precipitates attaching to the bacterial surface. The UV-C stress appears to enhance the strain’s biosorption capacity, as evidenced by the increased Cd mass percentage in the precipitates. These findings suggest that strain M1-B02 effectively removes Cd^2+^ through biosorption and that its adsorption capacity can be improved under UV-C exposure.

**Figure 4:**
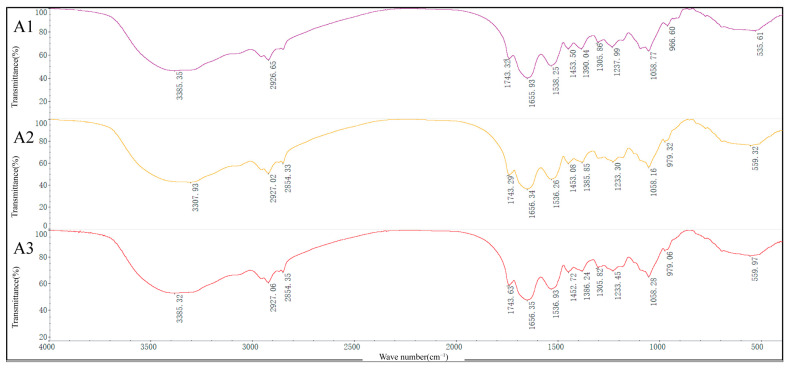
The effect on the ultrastructure of *Sphingomonas* sp. M1-B02 under FTIR. (**A1**) corresponds to untreated Sphingomonas sp. M1-B02. (**A2**) represents the bacterial cells after Cd^2+^ adsorption. (**A3**) shows the cells exposed to UV treatment followed by Cd^2+^ adsorption.

The main adsorption peaks of the infrared spectra of M1-B02 were analyzed; Figure 4 generated by FTIR spectroscopy shows the changes in vibration frequencies of experimental Groups A, B, and C cells under different treatments. The changes in these frequencies and their corresponding functional groups involved in Cd^2+^ binding are listed in Table 2. FTIR spectra showed the involvement of -CH or -NH in the adsorption of Cd^2+^ by stretching vibrations, -NO_2_ in the adsorption of Cd^2+^ by skeletal vibrations, CH_3_- in the adsorption of Cd^2+^ and thus C-H bending vibrations, aromatic ethers in the adsorption of Cd^2+^ by C-O stretching, RCH=CHR’ (trans) in the adsorption of Cd^2+^ by =C-H bending vibrations. 

### 3.4. The Genome Annotation of Sphingomonas sp. M1-B02

**Figure 5:**
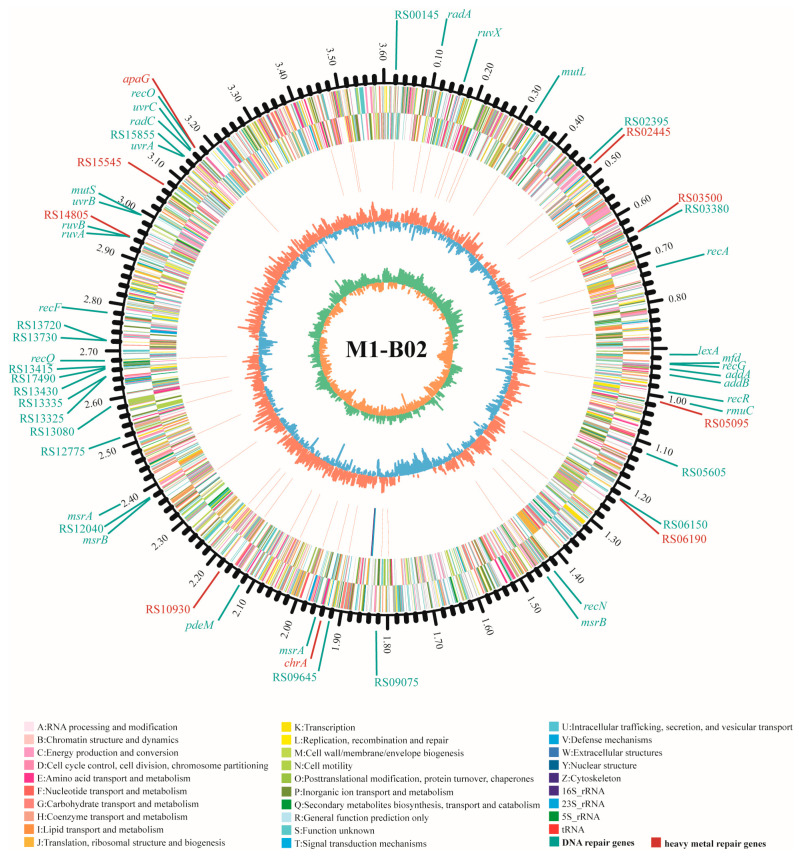
Genome circos of *Sphingomonas* sp. M1-B02 with DNA repair and heavy metal repair genes.

After whole genome sequencing of M1-B02, the coding genes were counted by comparison with different databases: 1649 genes were annotated according to the KEGG database, 2827 genes according to the COG database, 3306 genes according to the NR database, 2777 genes according to the Pfam database, 2108 genes according to the GO database (Figure S1). Numerous DNA protection or repair genes were annotated in the genome of strain M1-B02 to improve adaptation to oxidative stress induced by radiation and Cd stress (Figure 5 and Table S3). A total of 45 genes related to DNA repair and antioxidant were annotated, such as *mutL*, *mutS*, *uvrA*, *uvrC*, *recA*, *recF*, *recQ*, *recO*, *recR*, *radA*, *radC*, *addA*, *addB*, *recN*, *recF*, and *addA*. Genes numbered RS14805, RS03500, RS10930, RS02445, RS05095, RS06190, RS15545, RS16050, RS09730 were annotated related to heavy metal repair. From this, we can infer that when M1-B02 faces Cd^2+^ stress, the relevant functional genes participate in the redox process of Cd^2+^ and can transport Cd^2+^ out of the cell.

### 3.5. Analysis of Metabolites of Cd^2+^ Biosorption by Sphingomonas sp. M1-B02

**Figure 6:**
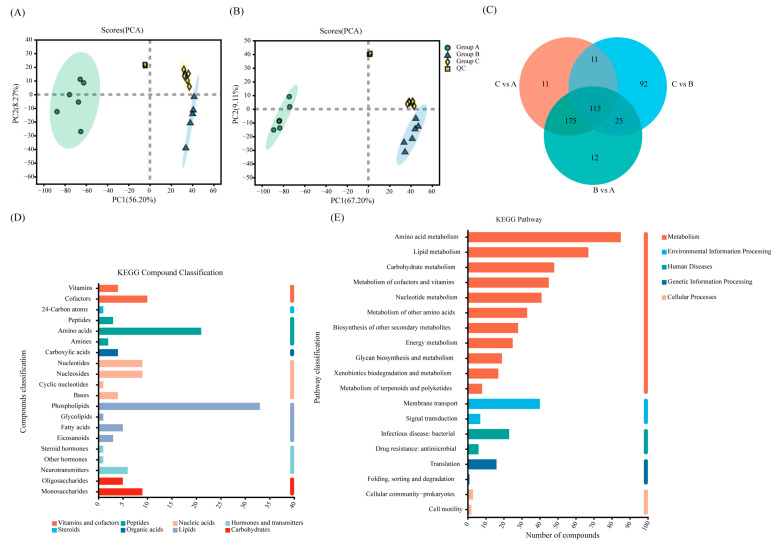
Metabolomics analysis of *Sphingomonas* sp. M1-B02 ((**A**,**B**), PCA scoring charts of metabolites; (**C**), Venn diagram of differential metabolites; (**D**), KEGG compound classification chart; (**E**), KEGG pathway statistics chart).

Principal Component Analysis (PCA) was performed on the metabolites of strain M1-B02 from different treatment groups A, B, and C to assess the similarity of samples within groups and the differences in samples between groups, to observe the trend of segregation between the two groups and to identify the pattern of metabolic changes related to Cd^2+^. The PCA scores are shown in Figure 6A,B. The quality control samples were tightly aggregated. The R^2^X (representing the explanatory power of the model for the X variables) of the PCA model was 56.2% and 67.2%, indicating that the data were well fitted, and the samples from each treatment group were separated, with the same characteristics, which indicated that Cd^2+^ had a significant effect on the metabolism of strain M1-B02, and all the samples were within the 95% confidence interval.

A total of 806 metabolites of the three groups of samples were detected and identified by LC-MS; 478 metabolites were annotated with KEGG Compound (https://www.kegg.jp/kegg/compound/) (accessed on 10 October 2023) [41] and classified according to the hierarchy of biological functions in which the metabolites are involved, and 751 metabolites were annotated with HMDB Compound (http://www.hmdb.ca) (accessed on 10 October 2023) [42]. The metabolites of the three groups of samples were annotated as mainly lipids, peptides, and nucleic acids (Figure 6C,D).

Based on the ID information of the metabolites compared to the KEGG compounds [43], information on the metabolic pathways involved in the metabolites was obtained, and analysis of the effects of metabolites on biological metabolic processes was performed, as shown in Figure 6E. The metabolites identified were annotated to five categories in the KEGG PATHWAY database, namely Metabolism, Human Diseases, Genetic Information Processing, Environmental Information Processing, and Cellular Processes. Most of the identified metabolites are involved in the Metabolism pathway, with the most involved in amino acid metabolism, metabolism of cofactors and vitamins, lipid metabolism, carbohydrate metabolism, and the second largest number of metabolites in nucleotide metabolism, and carbohydrate metabolism followed. 

## 4. Discussion

This study conducted a preliminary study on the adsorption capacity, adsorption process, and adsorption mechanism of experimental strains to 100 mg/L of Cd^2+^ within 24 h, and explored the adsorption changes in strains irradiated after UV-C irradiation. 

There is a correlation between different resistance mechanisms in microorganisms [44], e.g., physiological damage due to cellular desiccation is very similar to radiation damage, with double-strand breaks (DSBs) being a common feature implying that they can be repaired by common protein/enzymes [45], and reactive oxygen species (ROS) are produced by the cells during radiation stress, which are induced to be eliminated by catalase and peroxisomalase [46]. Desiccation leads to oxidative damage to cells [47], which can respond to desiccation and radiation stress through the same pathway. It has been shown that Cd^2+^ can cause damage to human cells, including alteration of protein conformation and thus cell cycle regulation, DNA repair, and DNA damage [48,49,50,51], and Cd can cause DNA single- and double-strand breaks [52]. For bacteria, heavy metals can cause DNA damage, which, in turn, affects the ability to process heavy metals, and the higher the concentration of heavy metals, the more severe the DNA damage in bacterial cells [53]. Cd can indirectly increase intracellular ROS clusters by inhibiting the activity of antioxidant enzymes such as superoxide dismutase, glutathione reductase, and glutathione peroxidase [54]; affecting the intracellular DNA damage response system, occupying the binding site of zinc and DNA polymerase and affecting the repair of DNA by DNA repair enzymes [55]; affecting the DNA cycle and apoptosis regulation by involvement in processes such as DNA methylation, thus increasing DNA instability and causing DNA damage [56]. It was demonstrated that exposure to Cd^2+^ induces oxidative stress in microorganisms, and *Sphingomonas* sp. responds to Cd^2+^ stress by enhancing the expression of proteins with antioxidant and detoxification properties [19]. *Sphingomonas* spp. are yellow-colored Gram-negative bacterium found in a variety of environments. The most expressed detoxification proteins in *Sphingomonas* sp. LK11, when exposed to Cd^2+^ stress, are detoxification proteins that are involved in cellular defense mechanisms; these detoxification proteins include chaperone proteins, heat shock response proteins, oxygen/free radical proteins, and stress response proteins, and in addition, oxidoreductase enzymes are also expressed, and many of these oxidoreductase enzymes are involved in stress-responsive processes [19].

Both Cd and UV-C irradiation cause oxidative stress in cells [26,57,58,59]. At a UV-C irradiation dose of 80 J/m^2^, the survival rate of strain M1-B02 was above 99%. Under the stimulation of this dose, it is speculated that the bacteria will increase the expression of antioxidant and DNA repair genes, increase the metabolism of antioxidant metabolites [57], protect the activity of the strain, and enhance its Cd^2+^ adsorption capacity. Numerous DNA protection or repair genes were annotated in the genome of strain M1-B02 to improve adaptation to oxidative stress induced by radiation and cadmium stress (Figure 4 and Table S1). For example, genes *mutL* and *mutS* play a role in DNA mismatch repair (MMR) [60], genes *uvrA* and *uvrC*, as well as *recA*, *recF*, *recQ*, *recO*, and *recR*, can participate in the recovery of DNA replication after UV irradiation [61,62], genes *radA* and *radC* can participate in the UV-induced DNA damage repair [63], genes *addA*, *addB* participate in DNA repair and recombination [64,65], the *recN* gene product is necessary for DNA repair and recombination, and the *recF* and *addA* genes provide overlapping activity [66]; gene numbered RS10715 (*pedM*) was annotated as “ligase-associated DNA damage response endonuclease PdeM”. In addition to this, through database comparison, we found that the genes numbered RS03500, and RS14805 were annotated as “heavy metal-binding domain-containing protein”, indicating that M1-B02 has a specific protein capable of binding Cd^2+^. Genes numbered RS02445, RS05095, RS06190, and RS15545 were annotated as “metalloregulator ArsR/SmtB family transcription factor”. ArsR family transcriptional regulators are widespread in bacteria and are involved in the detoxification of various metals [67]. Gene numbered RS10930 was annotated as “zinc transporter ZntB”; the mechanism of exocytosis of P-type ATPase is related to the genes *zntR*, *zntA*, and *zntB*, and the efflux of Znt can be activated by Cd^2+^ [68,69]. Genes numbered RS16050 (*apaG*) are described as “Co^2+^/Mg^2+^ efflux protein ApaG” and genes numbered RS09730 (*chrA*) are described as “chromate efflux transporter”; thus, it can be hypothesized that strain M1-B02 has the potential to treat other heavy metal ions.

Compared with other studies’ microbial adsorbents, in this study, M1-B02 has a higher adsorption efficiency (Table 3) and better potential for Cd^2+^ adsorption. When the pH value is 7 and the temperature is 30 °C, the strain has the highest adsorption capacity for Cd^2+^.

The pseudo-first-order kinetic model is widely used for the adsorption of biological adsorbents in liquids. In most biological metal adsorption processes, the pseudo-first-order kinetic model is not suitable for complete adsorption reactions, only applicable in the initial stage of the adsorption process [75]. Pseudo-second-order kinetic models can predict the complete process of biological adsorption. The results of the biosorption kinetic study showed that the pseudo-first-order model was the best fit with a higher correlation coefficient (R^2^) value than the pseudo-second-order model (R^2^ = 0.93) of 0.97, and the calculated data for the pseudo-first-order were closer to the experimental data (Figure 2 and Table 1). As can be seen from Table 1, the pseudo-first-order kinetic model and pseudo-second-order kinetic model both fit the adsorption of Cd^2+^ by strain M1-B02 well, and the equilibrium adsorption amounts calculated from the primary and secondary kinetics were 34.63 mg/g and 37.07 mg/g, respectively, which were not much different from the actual adsorption amount of 34.45 mg/g. This indicates that the process of the adsorption reaction is mainly chemisorption, which may be the result of the interaction between the functional groups on the cell surface and heavy metal ions, and thus, it is speculated that the adsorption of Cd^2+^ by this strain was mainly completed through ion exchange and complexation reactions on the bacterial surface. 

*Sphingomonas* spp. are yellow-colored Gram-negative bacterium found in a variety of environments. Electrostatic adsorption is a common strategy for heavy metal adsorption by microorganisms. Lipopolysaccharide (LPS) is currently considered to be a major component of the outer membrane of Gram-negative bacteria, which have a unique lipopolysaccharide and lipoprotein composition with anionic functional groups that give their surfaces a negative charge to bind metal cations to the cell membrane [76]. 

SEM-EDS analysis was performed, and strain M1-B02 adsorbed Cd^2+^ with precipitates and morphological changes on the surface. EDS analysis proved the presence of Cd^2+^ in these precipitates, indicating that the strain can remove Cd^2+^ by biosorption. FTIR spectra showed that the 3700–3300 cm^−1^ region is a characteristic region for O-H and N-H stretching vibrations, and the shifts in the frequencies from the regions 3385 to 3307 cm^−1^ indicate in Group B and A that it may be due to O-H or N-H stretching vibration absorption [77,78,79]. Hydroxyl groups are involved in metal–oxygen binding, as evidenced by the shift of the band to lower wave numbers, but after UV-C irradiation, O-H or N-H no longer participate in absorption;. It has been shown that hydroxyl groups have a high affinity for divalent cations [80,81], and these hydroxyl groups present in polysaccharides, in particular, can be negatively charged, thus promoting metal adsorption to a large extent [82]. The region between 1800 and 1500 cm^−1^ shows the characteristic bands of the protein, where 1700–1600 cm^−1^ is the amide-I band [83], and 1600–1500 cm^−1^ is a specific region of the amide-II band [79]; the shifts in the frequencies from the regions 1536–1538 cm^−1^ indicates that it may be -NO_2_ skeleton vibration absorption. The bands in the 1500–1200 cm^−1^ region are mainly from the C-H bending vibrations of -CH_3_, CH_2_, and -CH [84,85]; the transfer of 1390–1385 cm^−1^ region indicates the possible involvement of CH_3_-, possibly due to C-H bending vibration, while the transfer of 1237–1233 cm^−1^ region indicates the possible due to C-O stretching or P=O asymmetric stretching vibrations of PO_2_^−^ phosphodiesters. The peaks at 1220–1240 cm^−1^ correspond to symmetric scaling of the phospholipid moiety. The phosphate ester portion has chelating properties, and in phosphate-containing metabolites, the sugar phosphate esters play a crucial role in metal chelation [86,87]; the transfer of 966–979 cm^−1^ region indicates the involvement of RCH=CHR’(trans), possibly due to =C-H bending vibrations. The expansion and contraction of C-X can be seen from the frequency change of 535–559 cm^−1^. These suggest shifted bonds, and corresponding functional groups might be involved in Cd^2+^ biosorption. These indicate that heavy metal ions interact with chemical groups on the microbial surface (e.g., hydroxyl, amine, amino, etc.) to form metal complexes that are absorbed and immobilized on the cell surface. 

**Figure 7:**
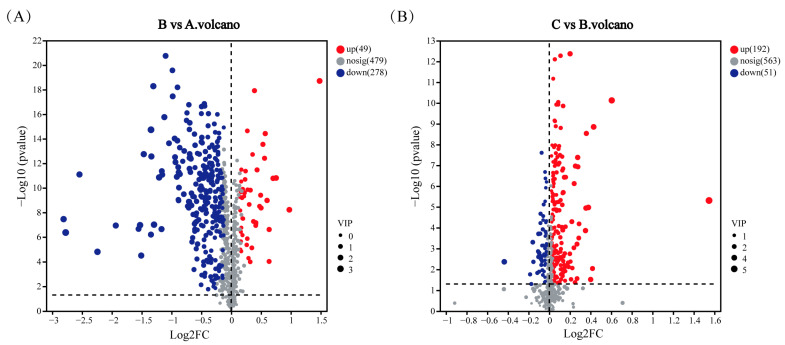
Volcano plots illustrating the differential metabolites identified in pairwise comparisons of groups (B vs. A and C vs. B). (**A**) Volcano plot of metabolites in the comparison between group B and group A. (**B**) Volcano plot of metabolites in the comparison between group C and group B.

Some bacteria precipitate heavy metal ions under heavy metal stress by releasing extracellular secretions such as polysaccharides, lipids, and proteins [88,89]. The mechanism of Cd^2+^ adsorption by the strain can be speculated by the analysis of differential metabolites and metabolic pathways. The LC-MS non-targeted metabolomics analysis identified a large variety of differential metabolites. As shown in Figure 7, differential metabolites were analyzed, with each point in the figure representing a specific metabolite and the size of the point indicating the VIP value. Blue represents the downregulated metabolites and red represents the upregulated metabolites; the more to the left and right and the upper side of the points, the more significant the difference in expression. Figure 7A shows that many metabolites showed significant changes under Cd^2+^ stress, and Cd^2+^ had a great effect on the metabolism of strain M1-B02, with 49 metabolites upregulated and 278 metabolites downregulated. Figure 7B shows that metabolites also changed upon Cd^2+^ adsorption by the strain after UV-C irradiation, with 192 metabolites upregulated and 51 metabolites downregulated. The metabolic pathway of Cd^2+^ adsorption by the strain may have changed after UV-C irradiation.

**Figure 8:**
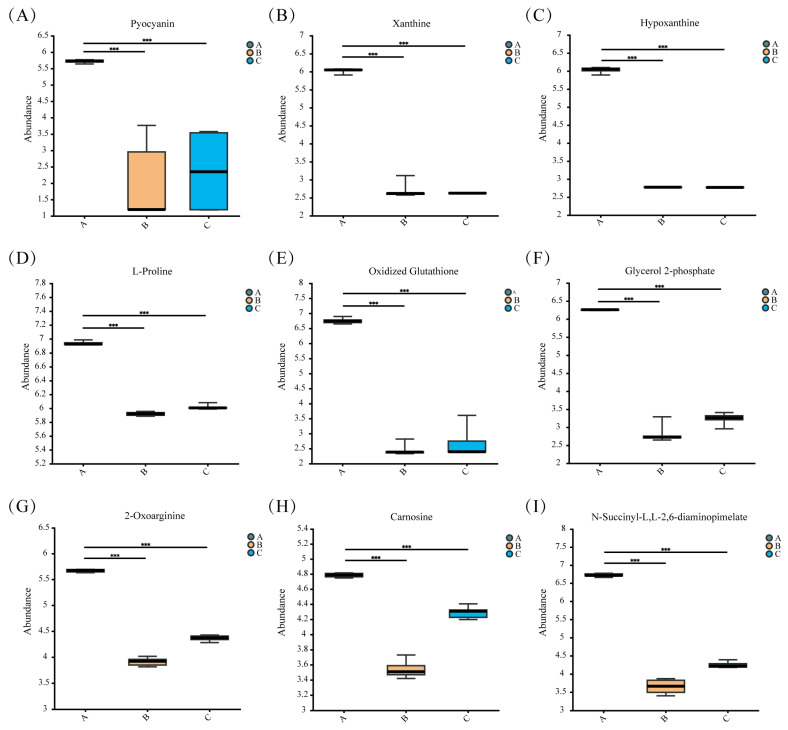
Box plot of distribution of significantly different metabolites in Group A, B, and C. The abundance of nine metabolites was compared across three groups (A, B, and C), with statistical significance indicated above each comparison. The boxplots represent the abundance values for (**A**) Pyocyanin, (**B**) Xanthine, (**C**) Hypoxanthine, (**D**) L-Proline, (**E**) Oxidized Glutathione, (**F**) Glycerol 2-phosphate, (**G**) 2-Oxoarginine, (**H**) Carnosine, and (**I**) N-Succinyl-L,L-2,6-diaminopimelate. Data points are visualized as boxplots, where the middle line represents the median, and the upper and lower bounds of the box correspond to the interquartile range (IQR). Groups are color-coded as A (grey), B (orange), and C (blue). *** represents *p* < 0.001.

Pyocyanin has a variety of biological activities such as antioxidant, antimicrobial, and immunomodulatory, and is an organic molecule with multiple functional groups, including carboxyl groups and nitrogen atoms, which give chlorophyllin the ability to chelate metal ions; pyocyanin has been reported to form complexes with heavy metal ions such as Cr^3+^, Ni^2+^, Cu^2+^, Zn^2+^ and Cd^2+^ [90]. Compared with the blank Group A, pyocyanin in Group B was downregulated by 0.35-fold (Figure 8A), which may be due to the formation of complexes with Cd^2+^. L-proline, a common amino acid, plays a critical role in protein synthesis and participates in various metabolic and physiological processes. It accumulates in response to numerous abiotic stresses and functions as a metal chelator to mitigate heavy metal stress. The carboxyl and amino groups in proline facilitate ligand binding with metal ions to form chelates [91,92]. In Group B, L-proline was downregulated by 0.14-fold compared to blank Group A (Figure 8D). Hypoxanthine and xanthine were downregulated 0.46-fold, and 0.45-fold, respectively, in Group B, presumably because hypoxanthine contains N-donors, which can act as Cd^2+^ complexes [93,94]. Glycerol 2-phosphate was downregulated 0.45-fold in Group B (Figure 8), probably due to bacterial cells that can take up metals mediated by phosphatase and glycerol-2-phosphate to release inorganic phosphate, allowing cadmium to precipitate as a cell-bound metal phosphate [95,96,97]. N-Succinyl-L,L-2,6-diaminopimelate was downregulated 0.54-fold in Group B and upregulated 1.16-fold in Group C compared to Group B. It was shown that the metals cadmium, cobalt, copper, cesium, manganese, thallium, and vanadium were significantly correlated with amino acid metabolic intermediates, including N-Succinyl-L,L-2,6- diaminopimelate, which is involved in tyrosine metabolism and lysine metabolism [98]. When N-Succinyl-L,L-2,6-diaminopimelate comes into contact with a Cd^2+^, the carbonyl oxygen atom of the carboxylic acid group and the nitrogen atom of the amino group may bind to vacancies in the cadmium ion to form a complex between cadmium and N-Succinyl-L,L-2,6-diaminopimelate. 2-oxoarginine, like N-succinyl-l,l-2,6-diaminopimelate, was significantly correlated with cadmium, copper, cesium, thallium and other metals as an intermediate in amino acid metabolism [98]. 2-oxoarginine is an amino acid derivative that contains a carboxyl group and two amino groups, and it is hypothesized that these functional groups could form complexes with Cd^2+^ or chemically react to immobilize Cd^2+^.

Pyocyanin, L-proline, hypoxanthine and xanthine, N-Succinyl-L,L-2,6-diaminopimelate, glycerol 2-phosphate were slightly upregulated in the UV-C irradiated Group C compared to Group B. It is speculated that this may be responsible for the increase in the Cd^2+^ adsorption capacity of the strain after UV-C irradiation. 

Bacterial cells can be oxidatively stressed under heavy metal stress, which can take place possibly by generating reactive oxygen species (ROS) such as superoxide radicals (O_2_^−^), singlet oxygen (^1^O_2_), hydrogen peroxide (H_2_O_2_) and hydroxyl radicals (OH^•^) [99], and production can rise significantly to three to four times [100]. Reducing agents (e.g., cysteine and glutathione) protect bacteria from metals [101]. Cadmium stress causes biological oxidative stress and a decrease in the ratio of reduced/oxidized glutathione occurs, with a 0.362-fold downregulation of oxidized glutathione in M1-B02 stressed by Cd^2+^ compared to the blank Group A [102]. UV-C irradiated Group C showed a 1.12-fold upregulation of carnosine compared to Group B. Carnosine, a naturally occurring dipeptide with antioxidant activity which inhibits protein carbonylation and glycoxidation [103,104,105], significantly inhibited the generation of reactive oxygen species (ROS) and 8-hydroxy-2′-deoxyguanosine (8-oxo-dG) [106], in addition to acid–base buffering activity [107], and metal ion chelating activity [108,109]. This may explain the good cadmium adsorption capacity of the strain.

## 5. Conclusions

In this study, we described a novel species of the genus *Sphingomonas* isolated from the moraine on the north slope of Mount Everest with high biosorption of Cd^2+^. The adsorption of *Sphingomonas* sp. M1-B02 for Cd^2+^ reached 68.90% in 24 h, and the adsorption after UV-C irradiation was enhanced to 80.56%. The genome of the strain was annotated with DNA repair genes such as *mutL*, *mutS*, *uvrA*, *uvrC*, *recA*, *recF*, *recQ*, *recO*, etc., and other heavy metal repair genes such as RS03500, RS14805, *apaG*, *chrA*, etc. A non-targeted metabolomic approach was used to study the changes in Cd^2+^-stressed bacterial metabolites, pyocyanin, proline, hypoxanthine and xanthine, N-Succinyl-L, L-2,6-diaminopimelate, glycerol 2-phosphate, which can form complexes with cadmium ions and immobilize cadmium ions. After UV-C irradiation, these metabolites were upregulated, which enhanced the adsorption capacity of the strain to Cd^2+^, while carnosine was upregulated, which enhanced the capacity of the strain to resist oxidative stress induced by Cd^2+^. 

*Sphingomonas* sp. M1-B02 has a great Cd^2+^ removal effect, owing to the comprehensive effects of cell membrane adsorption, intracellular complexation, intracellular ATPase efflux, and protection provided by metabolites. UV-C stress enhances the cadmium adsorption capacity of bacterial strains, which is a new insight into microbial adsorption of heavy metals and is instructive for the efficient microbial treatment of Cd^2+^-contaminated environments.

## Figures and Tables

**Table 1:** The kinetic fitting parameters of biosorption of Cd^2+^.

Type		Dynamic Parameters		
		$q_{e}$	$K_{x}\;$	$R^{2}$
Group B	First-order	34.63	1.83	0.97
	Second-order	37.07	0.07	0.93
Group C	First-order	40.18	1.47	0.98
	Second-order	43.05	0.05	0.95

Note: $K_{x}$ represents the rate constant for the respective kinetic model (pseudo-first-order or pseudo-second-order).

**Table 2:** The characteristic peak positions and functional groups of *Sphingomonas* sp. M1-B02.

Wave Number (cm^−1^)	Group Type (*v*)	Peak Intensity
3500–3300	Multimolecular association vO-H	S
	Carboxyl vO-H	VS
	Amide vN-H	Variable
1615–1510	-NO_2_	S
1380	-CH_3_	-
1275–1210	Aromatic ether	S
1000–650	σ_C-H_	Variable
1400–500	C-X	

Note: VS, and S are used to qualitatively indicate that the absorption intensity is very strong, strong.

**Table 3:** The absorption of Cd^2+^ by different microbial sorbents.

Bacteria	pH	Temperature (°C)	Concentration (mg/L)	Sorption Capacity (mg/g)	Reference
*Bacillus laterosporus*	7	25	1000	159.5	[70]
*Kocuria rhizophila*	8	35	150	9.07	[71]
*Sphingomonas* sp. LK11	-	28	-	44	[22]
*Paenibacillus* sp. LYX-1	8	30	100	30.68	[25]
*Cedecea* sp. SC19	7	37	500	126.19	[65]
*Bacillus cereus*	5	28	-	31.95	[72]
*Geobacillus toebii* sub.sp. *decanicus*	-	25	280	38.8	[73]
*Amanita rubescens*	5	20	-	27.3	[74]
*Sphingomonas* sp. M1-B02	7	30	100	34.45	

## Data Availability

The full-length 16S rRNA gene sequencing and genome data of strain S6-11T were stored in DDBJ/EMBL/GenBank with accession numbers ON527545.1 and CP110679, respectively.

## Supplementary Materials

The following supporting information can be downloaded at: https://www.mdpi.com/article/10.3390/microorganisms12122620/s1, Table S1: General genomic characteristics comparison of strains M1-B02, S2-65^T^, S5-59T, S8-45T, S9-5 ^T^, and their closely related species. Strains: 1. M1-B02;2. *S. asaccharolytica* DSM 10564^T^; 3. *S. panacisoli* HKS19^T^; 4. *S. pruni* NBRC 15498^T^; 5. *S. soli* DSM18313^T^; 6. *S. everestensis* S2-65^T^; 7. *S. qomolangmaensis* S5-59^T^; 8. *S. glaciei* S8-45^T^; 9. *S. radiodurans* S9-5 ^T^; Table S2: Comparison of genetic homology of strains M1-B02, S2-65T, S5-59T, S8-45T, S9-5 T, and their closely related species. Strains: 1. *S. asaccharolytica* DSM 10564^T^; 2. *S. panacisoli* HKS19^T^; 3. *S. pruni* NBRC 15498^T^; 4. M1-B02; 5. *S. soli* DSM 18313^T^; 6. *S. everestensis* S2-65^T^; 7. *S. qomolangmaensis* S5-59^T^; 8. *S. glaciei* S8-45^T^; 9. *S. radiodurans* S9-5^T^; Table S3: List of genes encoding antioxidant, DNA repair response, heavy metal repair proteins in the genomes of strain M1-B02; Table S4: Significantly changed metabolites in Cd^2+^ -stressed and UV irradiation *Sphingomonas* sp. M1-B02; Figure S1: Neighbor-joining phylogenetic tree based on 16S rRNA gene sequences of the strain S2-65^T^, S5-59^T^, M1-B02, S8-45^T^, S9-5^T^, and the type strains of other closely related species in the genus *Sphingomonas* and *Parasphingorhabdus*. *Parasphingorhabdus marina* FR1087^T^ (DQ781320) was used as an outgroup. The numbers on the tree indicate the percentages of bootstrap sampling derived from 1000 replications and the bootstrap values higher than 70% are shown. Bar, 0.01 substitutions per nucleotide position; Figure S2: Maximum-likelihood phylogenetic tree based on 16S rRNA gene sequences of the strain S2-65^T^, S5-59^T^, M1-B02, S8-45^T^, S9-5^T^, and the type strains of other closely related species in the genus *Sphingomonas* and *Parasphingorhabdus*. *Parasphingorhabdus marina* FR108^7T^ (DQ781320) was used as an outgroup. The numbers on the tree indicate the percentages of bootstrap sampling derived from 1000 replications and the bootstrap values higher than 70% are shown. Bar, 0.02 substitutions per nucleotide position; Figure S3: Minimum-evolution phylogenetic tree based on 16S rRNA gene sequences of the strain S2-65^T^, S5-59^T^, M1-B02, S8-45^T^, S9-5^T^, and the type strains of other closely related species in the genus *Sphingomonas* and *Parasphingorhabdus*. *Parasphingorhabdus* marina FR1087T (DQ781320) was used as an outgroup. The numbers on the tree indicate the percentages of bootstrap sampling derived from 1000 replications and the bootstrap values higher than 70% are shown. Bar, 0.01 substitutions per nucleotide position; Figure S4: Polar lipids profile of strain M1-B02. Total lipids were visualized after two-dimensional TLC and applying 5% ethanolic molybdatophosphoric acid. The solvent system was phosphomolybdic acid (A), molybdenum blue (B), indigohydrone (C), and α-naphthol (D) from left to right and top to bottom; Figure S5: Clustering heat graph and VIP bar chart (The right side is the VIP bar chart of metabolites. The bar length indicates the contribution value of the metabolites to the difference between the two groups. The default value is not less than 1. The larger the value, the greater the difference between the two groups. The bar color represents the significant difference in metabolites between the two groups of samples, namely the *p* value. The smaller the *p* value, the larger the −log10 (*p* value), and the darker the color. On the right * represents *p* < 0.05, ** represents *p* < 0.01, and *** represents *p* < 0.001).
