# Supplementary material for: UV-C Exposure Enhanced the Cd2+ Adsorption Capability of the Radiation-Resistant Strain Sphingomonas sp. M1-B02
Source: Microorganisms. 2024 Dec 18;12(12):2620. doi: 10.3390/microorganisms12122620 (PMC11678681; doi:10.3390/microorganisms12122620)
Supplement: Supplementary file 1 [file microorganisms-12-02620-s001.zip › microorganisms-3345035-supplementary.pdf]

Supplementary Materials

Table S1. Comparison of the general genomic characteristics of strains M1-B02, S2-65^{T}, S5-59^{T}, S8-45^{T}, S9-5^{T}, and their closely related species.

Strains: 1. M1-B02;2. *S. asaccharolytica* DSM 10564^{T}; 3. *S. panacisoli* HKS19^{T}; 4. *S. pruni* NBRC 15498^{T}; 5. *S. soli* DSM18313^{T}; 6. *S. everestensis* S2-65^{T}; 7.*S. qomolangmaensis* S5-59^{T}; 8. *S. glaciei* S8-45^{T}; 9. *S. radiodurans* S9-5^{T}.

Characteristics	1	2	3	4	5	6	7	8	9
**Genome size (bp)**	**3605070**	4608803	3403025	4482342	3514102	3828410	3429145	2880162	3678193
**Contig N50**	**3605070**	50453	3403025	50291	278470	3797930	3429145	2880162	3678193
**CDS**	**3488**	4682	3473	4243	3423	3583	3213	2813	3528
**RNA**	**60**	52	51	50	51	65	57	53	57
**rRNA**	**3**	3	3	3	3	9	6	3	6
**tRNA**	**45**	46	45	44	45	53	48	47	48
**5S rRNA**	**1**	1	1	1	1	3	2	1	2
**16S rRNA**	**1**	1	1	1	1	3	2	1	2
**23S rRNA**	**1**	1	1	1	1	3	2	1	2
**GI**		3	4				6	6	
**KEGG**	**1649**	1890	1528	1858	1628	1720	1763	1424	1675
**COG**	**2827**	2956	2204	2921	2337	2887	2546	2273	2903
**NR**	**3306**	4291	3043	4110	3349	3454	3076	2811	3258
**Pfam**	**2777**	3539	2665	3524	2723	2877	2617	2271	2863
**GO**	**2108**	-	-	-	-	2012	1379	574	2309
**Swiss-Prot**	**2317**	142	1039	1234	1099	2363	2147	1874	2372
**CAZyme**	**141**	189	100	138	99	137	129	76	139
**Secondary metabolite**	**3**	7	3	15	3	4	6	2	2
**DNA G+C content (mol%)**	**65.6**	64.8	65.1	65.4	65.1	66.1	66.4	66.6	65.8
**GenBank accession number**	**GCA_026167525.1**	GCA_001598355.1	GCA_007859635.1	GCA_001598455.1	GCA_001591025.1	GCA_021513175.1	GCA_024496245.1	GCA_023380025.1	GCA_020866845.1

* Note: **Genome size (bp)**: The total size of the genome in base pairs (bp), representing the length of the complete DNA sequence; **Contig N50**: A metric for genome assembly quality, representing the contig length at which 50% of the total genome assembly is covered; **CDS**: Coding sequences (number of predicted coding sequences in the genome); RNA: Total number of RNA genes, including all rRNA (ribosomal RNA) and tRNA (transfer RNA) present in the genome; **rRNA**: The number of ribosomal RNA genes, typically categorized into 5S, 16S, and 23S rRNA; **tRNA**: The number of transfer RNA genes, which are involved in protein synthesis by transporting amino acids to the ribosome; **5S rRNA**: The number of 5S ribosomal RNA genes, a component of the large ribosomal subunit that plays a structural role in ribosome function; **16S rRNA**: The number of 16S ribosomal RNA genes, widely used in phylogenetic studies as a marker for bacterial identification; **23S rRNA**: The number of 23S ribosomal RNA genes, part of the large ribosomal subunit involved in the peptidyl transferase activity of the ribosome; **GI**: The number of genomic islands (GI), which are regions of the genome acquired through horizontal gene transfer, often containing genes related to virulence or adaptation; **KEGG**: The number of genes annotated in the Kyoto Encyclopedia of Genes and Genomes (KEGG) database, representing metabolic pathways and functional modules; **COG**: The number of genes categorized into Clusters of Orthologous Groups (COG), which classify proteins based on their evolutionary relationships; **NR**: The number of genes annotated in the NCBI non-redundant protein database, representing unique protein sequences; **Pfam**: The number of protein families identified based on conserved domains from the Pfam database; **GO**: The number of genes assigned Gene

Ontology (GO) terms, which describe gene products in terms of their associated biological processes, cellular components, and molecular functions; **Swiss-Prot**: The number of genes annotated in the manually curated Swiss-Prot protein database, which provides high-quality functional information; **CAZyme**: The number of carbohydrate-active enzymes (CAZymes) identified, which are involved in the synthesis, modification, and degradation of carbohydrate; **Secondary metabolite**: The number of biosynthetic gene clusters identified for secondary metabolites, which are compounds not essential for growth but often important for ecological interactions; **DNA G+C content (mol%)**: The percentage of guanine (G) and cytosine (C) bases in the genome, a key feature of genomic composition; **GenBank accession number**: The unique identifier assigned to the genome sequence in the GenBank database, used for referencing and retrieving the sequence.

Table S2. Comparison of genetic homology of strains M1-B02, S2-65^{T}, S5-59^{T}, S8-45^{T}, S9-5^{T}, and their closely related species.

Strains: 1, *S. asaccharolytica* DSM 10564^{T}; 2, *S. panacisoli* HKS19^{T}; 3, *S. pruni* NBRC 15498^{T}; 4, M1-B02; 5, *S. soli* DSM 18313^{T}; 6, *S. everestensis* S2-65^{T}; 7, *S. qomolangmaensis* S5-59^{T}; 8, *S. glaciei* S8-45^{T}; 9, *S. radiodurans* S9-5^{T}.

Strain		1	2	3	4	5	6	7	8	9
	1	*								
	2	73.27	*							
	3	73.44	78.92	*						
**ANIb (%)**	4	73.21	87.25	78.48	*					
	5	77.09	72.95	73.08	73.07	*				
	6	77.06	73.03	73.14	73.04	75.78	*			
	7	74.49	73.68	73.72	73.55	74.09	74.26	*		
	8	70.72	70.62	70.45	70.42	70.33	70.58	70.43	*	
	9	73.36	73.43	73.70	73.49	72.56	72.89	73.37	70.24	*
	1	*								
	2	83.53	*							
	3	83.57	84.33	*						
**ANIm (%)**	4	83.69	89.04	84.38	*					
	5	84.00	83.61	83.84	83.70	*				
	6	83.96	83.71	83.75	83.83	84.07	*			
	7	83.63	83.43	83.31	83.37	83.40	83.60	*		
	8	83.22	83.33	83.39	83.83	83.25	83.47	83.36	*	
	9	83.62	83.66	83.69	83.72	83.55	83.81	83.44	83.22	*
	1	*								
	2	74.47	*							
	3	74.36	79.49	*						
**OrthoANI (%)**	4	74.49	88.03	79.44	*					
	5	77.97	73.96	74.09	73.94	*				
	6	78.36	74.22	74.29	74.12	76.61	*			
	7	75.33	74.52	74.49	74.24	74.69	75.17	*		
	8	72.02	71.63	71.43	71.61	71.53	72.06	71.93	*	
	9	74.45	74.01	74.17	74.02	73.39	73.75	74.20	71.37	*
	1	*								
	2	19.80	*							
	3	19.30	24.80	*						
**DDH (%)**	4	19.80	35.30	22.30	*					
	5	24.60	19.70	19.30	20.20	*				
	6	21.40	20.10	19.50	20.20	20.50	*			
	7	19.90	19.70	19.50	19.40	19.70	20.00	*		
	8	14.10	19.10	19.10	19.20	19.80	19.80	18.80	*	
	9	19.60	20.10	19.80	19.50	20.10	19.80	19.50	19.10	*
	1	*								
	2		*							
	3	79.72	75.86	*						
**AAI (%)**	4	88.36	84.87	72.40	*					
	5	74.56	76.98	78.34	81.32	*				
	6	77.90	77.20	78.22	85.78	76.15	*			
	7	80.50	63.86	63.91	62.53	80.85	81.22	*		
	8	82.48	75.30	76.66	79.34	79.14	84.34	76.55	*	
	9	76.39	76.98	78.98	83.83	69.49	79.68	84.33	75.69	*

* Note: **ANIb (%):** Average Nucleotide Identity based on BLAST (ANIb) is a measure of genetic similarity between two genomes calculated using the BLAST algorithm. It reflects the overall percentage of nucleotide similarity across shared genomic regions; **ANIm (%):** Average Nucleotide Identity based on MUMmer (ANIm) calculates genomic similarity using the MUMmer alignment tool. Like ANIb, it provides a percentage similarity between two genomes but uses a different algorithm for alignment; **OrthoANIu (%):** Orthologous Average Nucleotide Identity using USEARCH (OrthoANIu) is another metric to evaluate genomic similarity between two genomes, specifically focusing on orthologous sequences. It is calculated using the USEARCH tool; **DDH (%):** Digital DNA-DNA Hybridization (DDH) is an in silico method used to estimate the relatedness between two genomes. It is expressed as a percentage and serves as an alternative to traditional wet-lab DNA-DNA hybridization experiments; **AAI (%):** Average Amino Acid Identity (AAI) measures the average similarity of amino acid sequences

between orthologous genes shared by two genomes. It is expressed as a percentage and is used to assess genetic relatedness at the protein level.

Table S3. List of genes encoding antioxidant, DNA repair response, heavy metal repair proteins in the genomes of strain M1-B02.

Locus tag	Gene Name	Symbol	Locus tag	Gene Name	Symbol
RS04680	ATP-dependent DNA helicase RecG	*recG*	RS13720	ligase-associated DNA damage response exonuclease	
RS13415	cisplatin damage response ATP-dependent DNA ligase		RS12040	MmcB family DNA repair protein	
RS09075	cold-shock protein		RS07425	peptide-methionine (R)-S-oxide reductase MsrB	*msrB*
RS17490	cold-shock protein		RS12070	peptide-methionine (R)-S-oxide reductase MsrB	*msrB*
RS15855	cold-shock protein		RS09800	peptide-methionine (S)-S-oxide reductase MsrA	*msrA*
RS13560	DNA helicase RecQ	*recQ*	RS12175	peptide-methionine (S)-S-oxide reductase MsrA	*msrA*
RS01735	DNA mismatch repair endonuclease MutL	*mutL*	RS13325	putative DNA modification/repair radical SAM protein	
RS15090	DNA mismatch repair protein MutS	*mutS*	RS02395	recombinase family protein	
RS05040	DNA recombination protein RmuC	*rmuC*	RS05605	recombinase family protein	
RS00510	DNA repair protein RadA	*radA*	RS13080	recombinase family protein	
RS15970	DNA repair protein RadC	*radC*	RS03790	recombinase RecA	*recA*
RS07265	DNA repair protein RecN	*recN*	RS05030	recombination mediator RecR	*recR*
RS16025	DNA repair protein RecO	*recO*	RS06150	RecX family transcriptional regulator	
RS14010	DNA replication/repair protein RecF	*recF*	RS12775	SOS response-associated peptidase	
RS00145	DNA starvation/stationary phase protection protein		RS04600	transcriptional repressor LexA	*lexA*
RS13430	DNA starvation/stationary phase protection protein		RS04690	transcription-repair coupling factor	*mfd*
RS04770	double-strand break repair helicase AddA	*addA*	RS09645	UvrB/UvrC motif-containing protein	
RS04775	double-strand break repair protein AddB	*addB*	RS03380	UvrD-helicase domain-containing protein	
RS13335	error-prone DNA polymerase		RS14805	heavy metal-binding domain-containing protein	
RS15900	excinuclease ABC subunit UvrA	*uvrA*	RS03500	heavy-metal-associated domain-containing protein	
RS15070	excinuclease ABC subunit UvrB	*uvrB*	RS10930	zinc transporter ZntB	
RS16005	excinuclease ABC subunit UvrC	*uvrC*	RS02445	metalloregulator ArsR/SmtB family transcription factor	
RS14830	Holliday junction branch migration DNA helicase RuvB	*ruvB*	RS05095	metalloregulator ArsR/SmtB family transcription factor	
RS14820	Holliday junction branch migration protein RuvA	*ruvA*	RS06190	metalloregulator ArsR/SmtB family transcription factor	
RS00865	Holliday junction resolvase RuvX	*ruvX*	RS15545	metalloregulator ArsR/SmtB family transcription factor	
RS13730	ligase-associated DNA damage response DEXH box helicase		RS16050	Co2+/Mg2+ efflux protein ApaG	*apaG*
RS10715	ligase-associated DNA damage response endonuclease PdeM	*pdeM*	RS09730	chromate efflux transporter	*chrA*

Table S4. Significantly changed metabolites in Cd^{2+}-stressed and UV irradiation *sphingomonas* sp. M1-B02

Metabolite	Regulate	FC(B/A)	KEGG Compound ID	Formula	VIP	P_value
6-Thioguanosine	up	2.7928	-	C10H13N5O4S	2.2816	1.94E-19
Pyocyanin	down	0.3514	C01748	C13H10N2O	2.6028	3.19E-05
S-Acetyldihydrolipoamide-E	down	0.2113	C16255	C10H19NO2S2	2.8317	1.59E-05
7-Aminomethyl-7-carbaguanine	down	0.1461	C16675	C7H9N5O	3.1589	4.34E-07
Dephospho-CoA	down	0.4078	C00882	C21H35N7O13P2S	2.8319	9.89E-08
DHAP(8:0)	down	0.2615	-	C11H21O7P	2.5419	1.17E-07
Pantothenic Acid	down	0.5448	C00864	C9H17NO5	2.2937	9.50E-11
N-Acetyl-L-Glutamic Acid	down	0.3955	C00624	C7H11NO5	2.6218	2.75E-13
Oxidized Glutathione	down	0.362	C00127	C20H32N6O12S2	2.6692	1.79E-13
Glycerol 2-phosphate	down	0.4487	C02979	C3H9O6P	2.3864	8.44E-12
Glutathione, oxidized	down	0.4327	C00127	C20H32N6O12S2	2.7718	1.41E-11
2-Keto-6-acetamidocaproate	down	0.5402	C05548	C8H13NO4	2.2982	2.07E-12
Thidiazuron	down	0.5349	C18812	C9H8N4OS	2.2841	1.41E-14
UDP-D-Galactose	down	0.5067	C00052	C15H24N2O17P2	2.3486	3.49E-18
Leucyl-Lysine	down	0.4045	-	C12H25N3O3	2.6569	5.18E-19
2,3-Diaminopyridine	down	0.4666	-	C5H7N3	2.314	1.76E-21
S-Hydroxymethylglutathione	down	0.5182	C14180	C11H19N3O7S	2.3266	9.52E-15
Caffeoyl tyrosine	down	0.3938	-	C18H17NO6	3.1239	1.84E-15
Hypoxanthine	down	0.4599	C00262	C5H4N4O	2.5501	1.72E-16
N-Succinyl-L,L-2,6-diaminopimelate	down	0.5432	C04421	C11H18N2O7	2.4727	5.45E-12
Cefminox	down	0.5205	-	C16H21N7O7S3	2.339	3.12E-13
Pyro-L-glutaminyl-L-glutamine	down	0.5315	-	C10H15N3O5	2.4368	1.26E-11
Gamma-Glutamylalanine	down	0.5233	C03740	C8H14N2O5	2.3482	8.09E-13
LysoPE(P-16:0/0:0)	down	0.1715	-	C21H44NO6P	2.6112	8.07E-12
Xanthine	down	0.4464	C00385	C5H4N4O2	2.3478	4.30E-12
N-Palmitoyl Tryptophan	down	0.1426	-	C27H42N2O3	2.7544	3.51E-08
Hexadecanedioic acid	down	0.3407	C19615	C16H30O4	2.3671	2.21E-07
2-Amino-3-methylbenzoate	down	0.3935	C16571	C8H9NO2	2.4601	6.36E-07
N-Acetylmuramate	down	0.3468	C02713	C11H19NO8	2.811	1.08E-07
Nalpha-Acetyl-L-arginine	down	0.4449	-	C8H16N4O3	2.5315	2.28E-07

Metabolite	Regulate	FC(C/B)	KEGG Compound ID	Formula	VIP	P value
6-Thioguanosine	up	1.0562	-	C10H13N5O4S	1.7697	3.95E-08
Xylobiose	up	1.019	C01630	C10H18O9	1.1548	1.07E-08
Nalpha-Acetyl-L-arginine	up	1.3367	-	C8H16N4O3	2.9254	0.00906
L-beta-aspartyl-L-leucine	up	1.0983	-	C10H18N2O5	2.0512	0.004004
Gamma-Glu-Cys	up	1.0353	C00669	C8H14N2O5S	1.2354	0.007353
Morph	up	1.0932	C01516	C10H16N2O4	2.2163	0.000133
Cysteineglutathione disulfide	down	0.9602	-	C13H22N4O8S2	1.6384	2.76E-05
Thidiazuron	up	1.347	C18812	C9H8N4OS	3.8348	1.41E-09
Dihydrofolic acid	up	1.0812	C00415	C19H21N7O6	1.7309	0.01731
S-Adenosylhomocysteine	down	0.9782	C00021	C14H20N6O5S	1.199	0.000183
UDP-D-Galactose	up	1.0963	C00052	C15H24N2O17P2	1.5946	0.01527
Vomifoliol	up	1.2215	C04166	C13H20O3	2.5793	6.56E-05
Phosphovanillin	up	1.1142	-	C8H9O6P	1.7472	0.01214
Dethiobiotin	up	1.0657	C01909	C10H18N2O3	1.7097	0.003581
Imidazolepropionic acid	up	1.0468	C20522	C6H8N2O2	1.3481	0.005947
4-Hydroxycitrulline	up	1.1388	-	C6H13N3O4	2.2101	0.007601
Citrulline	up	1.2047	C00327	C6H13N3O3	2.8357	0.000615
Glycerol 2-phosphate	up	1.1532	C02979	C3H9O6P	1.9943	0.004214
Cytidine diphosphate (CDP)	up	1.2101	-	C9H15N3O11P2	3.3069	4.17E-08
Pantothenic Acid	up	1.2753	C00864	C9H17NO5	3.3344	0.000139
P-Coumaric acid	up	1.0615	C00811	C9H8O3	1.3714	0.0415
DHAP(8:0)	up	2.923	-	C11H21O7P	5.5222	4.93E-06
2-Keto-6-acetamidocaproate	up	1.1121	C05548	C8H13NO4	1.7788	0.03474
Codeine-6-glucuronide	up	1.1095	C16577	C24H29NO9	2.163	0.007907
L-2-Aminoethyl seryl phosphate	up	1.1152	C03872	C5H13N2O6P	2.2388	0.006748
4-(Glutamylamino) butanoate	up	1.0991	C15767	C9H16N2O5	1.9505	0.009631
N-Succinyl-L,L-2,6-diaminopimelate	up	1.1635	C04421	C11H18N2O7	2.8281	5.13E-05
Cefminox	up	1.521	-	C16H21N7O7S3	4.6598	7.46E-11
Pyro-L-glutaminyl-L-glutamine	up	1.1573	-	C10H15N3O5	2.2202	0.02983
Gamma-Glutamylalanine	up	1.1797	C03740	C8H14N2O5	2.5711	0.000924

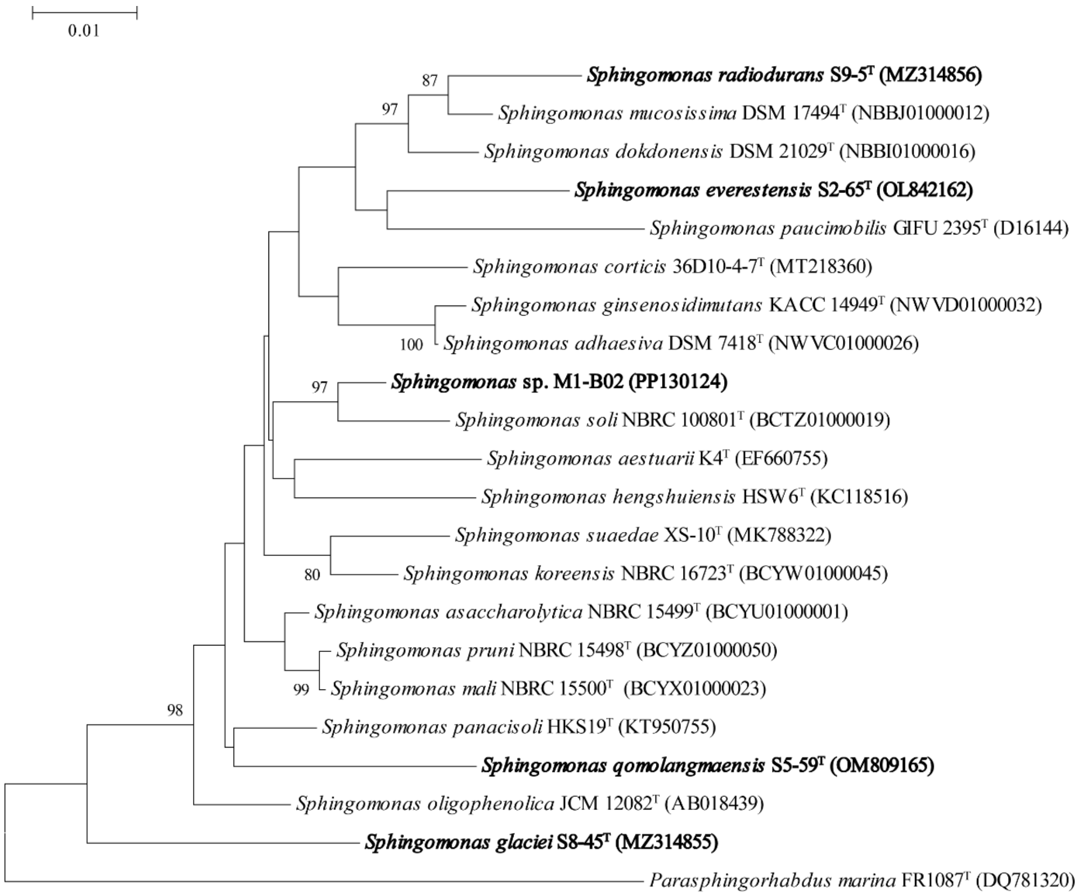

Figure S1. Neighbor-joining phylogenetic tree based on 16S rRNA gene sequences of the strain S2-65^{T}, S5-59^{T}, M1-B02, S8-45^{T}, S9-5^{T}, and the type strains of other closely related species in the genus *Sphingomonas* and *Parasphingorhabdus*. *Parasphingorhabdus marina* FR1087^{T} (DQ781320) was used as an outgroup. The numbers on the tree indicate the percentages of bootstrap sampling derived from 1000 replications and the bootstrap values higher than 70% are shown. Bar, 0.01 substitutions per nucleotide position.

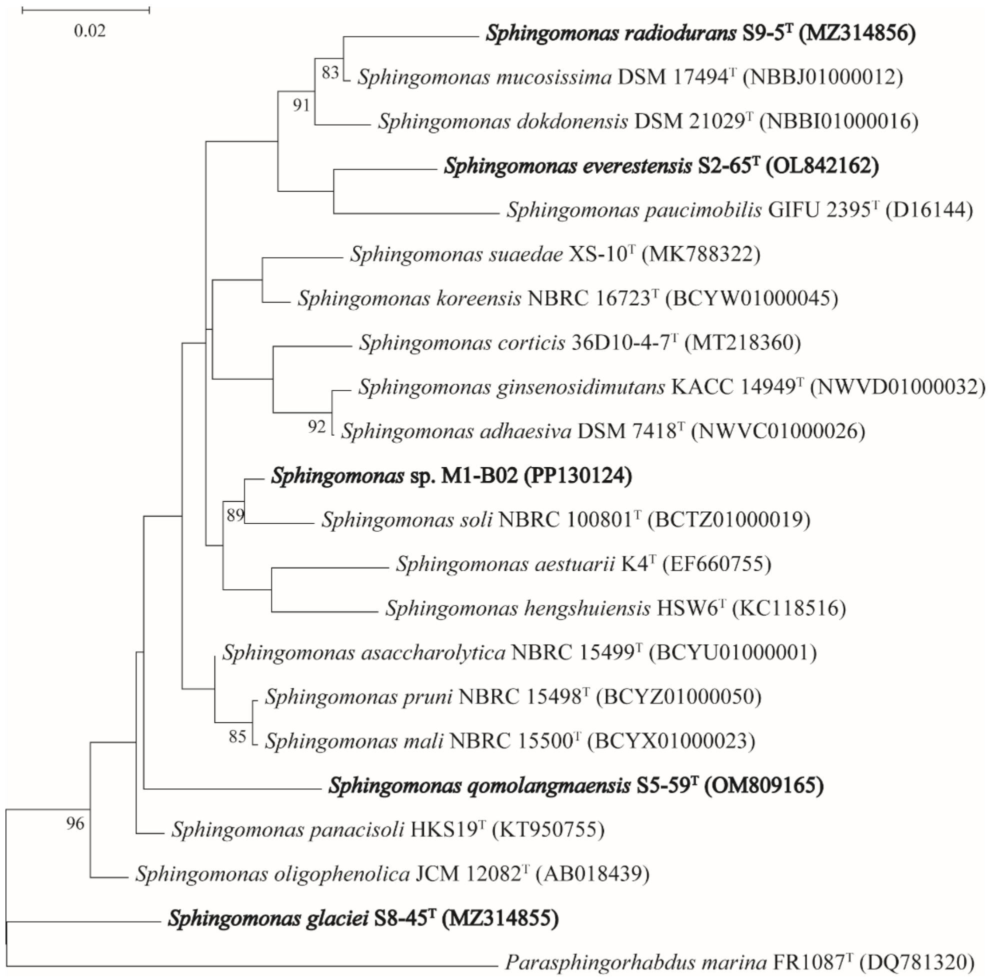

Figure S2. Maximum-likelihood phylogenetic tree based on 16S rRNA gene sequences of the strain S2-65^{T}, S5-59^{T}, M1-B02, S8-45^{T}, S9-5^{T}, and the type strains of other closely related species in the genus *Sphingomonas* and *Parasphingorhabdus*. *Parasphingorhabdus marina* FR1087^{T} (DQ781320) was used as an outgroup. The numbers on the tree indicate the percentages of bootstrap sampling derived from 1000 replications and the bootstrap values higher than 70% are shown. Bar, 0.02 substitutions per nucleotide position.

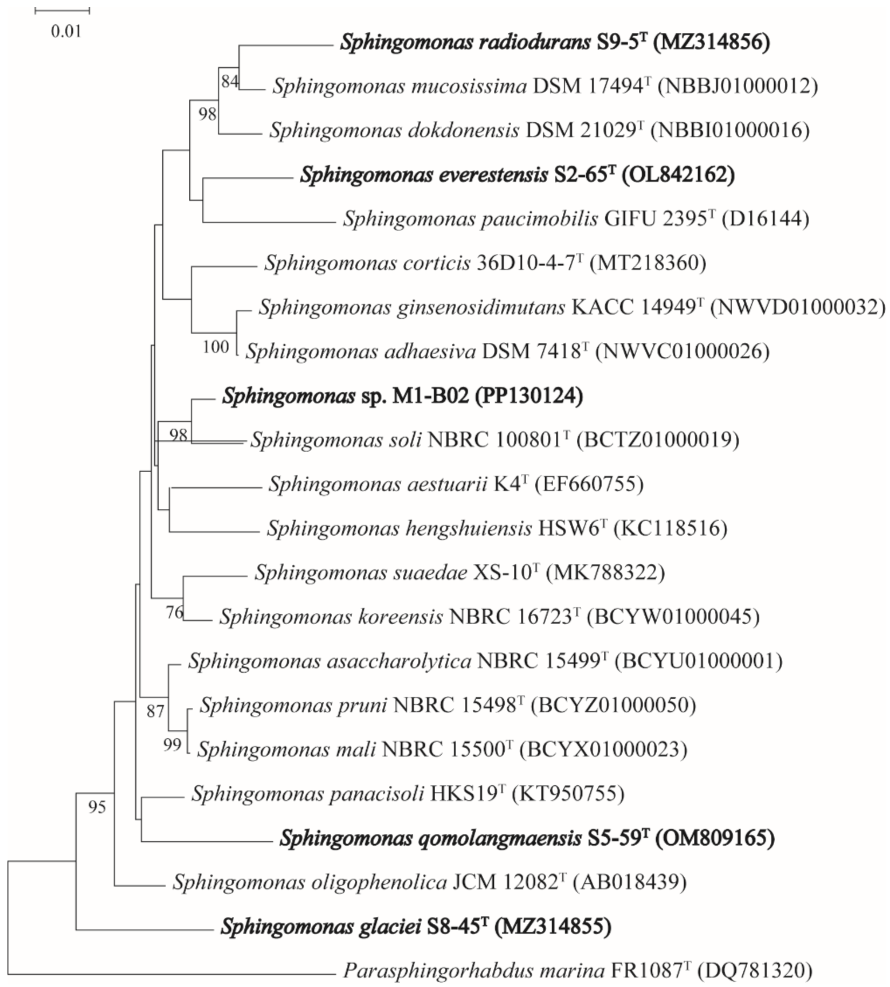

Figure S3. Minimum-evolution phylogenetic tree based on 16S rRNA gene sequences of the strain S2-65^T, S5-59^T, M1-B02, S8-45^T, S9-5^T, and the type strains of other closely related species in the genus *Sphingomonas* and *Parasphingorhabdus*. *Parasphingorhabdus marina* FR1087^T (DQ781320) was used as an outgroup. The numbers on the tree indicate the percentages of bootstrap sampling derived from 1000 replications and the bootstrap values higher than 70% are shown. Bar, 0.01 substitutions per nucleotide position.

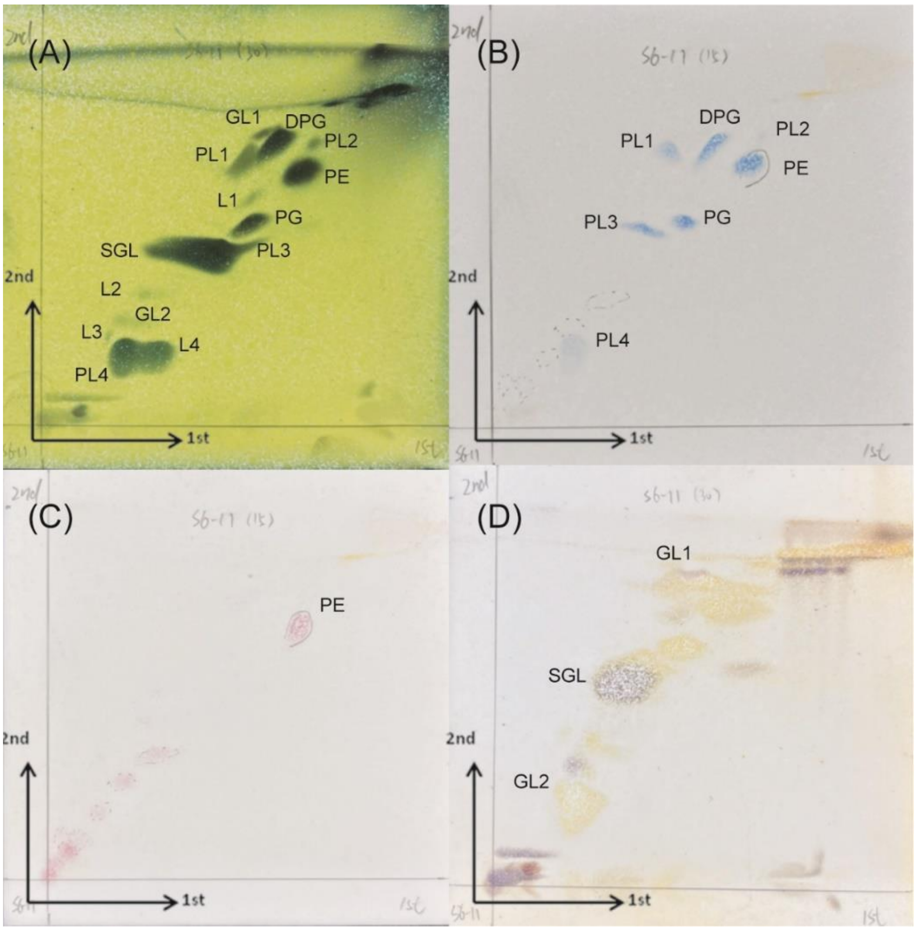

Figure S4. Polar lipids profile of strain M1-B02. Total lipids were visualized after two-dimensional TLC and applying 5% ethanolic molybdatophosphoric acid. The solvent system was phosphomolybdic acid (**A**), molybdenum blue (**B**), indigohydrone (**C**), and α-naphthol (**D**) from left to right and top to bottom.

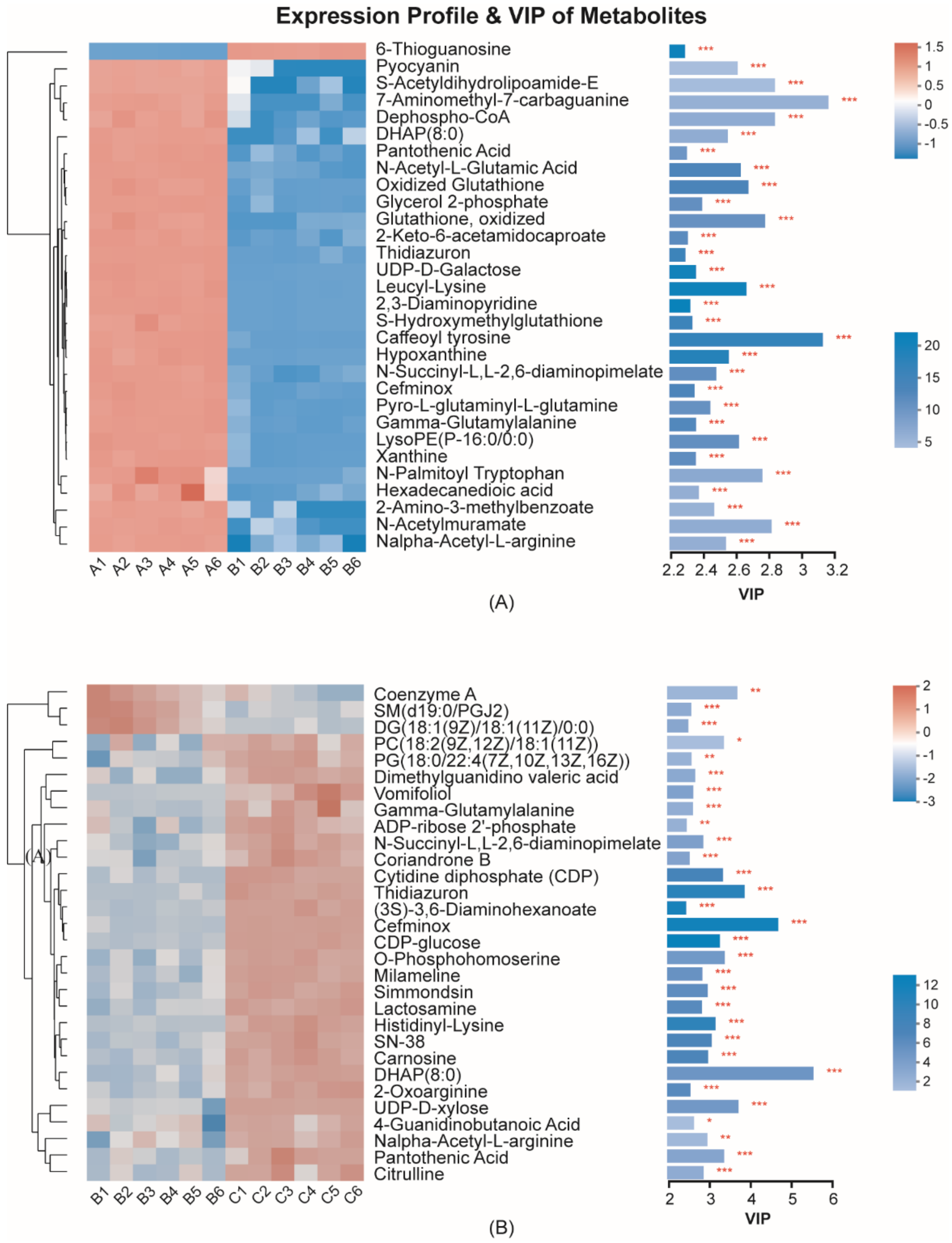

Figure S5. Clustering heat graph and VIP bar chart. The right side is the VIP bar chart of metabolites. The bar length indicates the contribution value of the metabolites to the difference between the two groups. The default value is not less than 1. The larger the value, the greater the difference between the two groups. The bar color represents the significant difference in metabolites between the two groups of samples, namely the *P* value value. The smaller the *P* value, the larger the - log10 (*P* value), and the darker the color. On the right * represents $P < 0.05$, * * represents $P < 0.01$, and * * * represents $P < 0.001$
